# Polychlorinated Biphenyls, Lead, and Mercury Are Associated with Liver Disease in American Adults: NHANES 2003–2004

**DOI:** 10.1289/ehp.1002720

**Published:** 2010-09-03

**Authors:** Matt Cave, Savitri Appana, Mihir Patel, Keith Cameron Falkner, Craig J. McClain, Guy Brock

**Affiliations:** 1 Department of Medicine, Division of Gastroenterology, Hepatology, and Nutrition; 2 Department of Pharmacology and Toxicology; 3 Robley Rex Veterans Affairs Medical Center and; 4 Department of Bioinformatics and Biostatistics, University of Louisville, Louisville, Kentucky, USA

**Keywords:** environmental liver disease, hepatotoxicity, lead, mercury, NAFLD, NASH, nonalcoholic fatty liver disease, nonalcoholic steatohepatitis, PCBs, polychlorinated biphenyls, TASH

## Abstract

**Background:**

High-level occupational exposures to some industrial chemicals have been associated with liver diseases, including nonalcoholic fatty liver disease (NAFLD). However, the potential role of low-level environmental pollution on liver disease in the general population has not been evaluated.

**Objective:**

We determined whether environmental pollutants are associated with an elevation in serum alanine aminotransferase (ALT) activity and suspected NAFLD in U.S. adults.

**Methods:**

This cross-sectional cohort study evaluated adult participants without viral hepatitis, hemochromatosis, or alcoholic liver disease from the National Health and Nutrition Examination Survey (NHANES) for 2003–2004. ALT elevation was defined in men as ≥ 37 IU/L (age18–20 years) and ≥ 48 IU/L (age ≥ 21 years) and in women as ≥ 30 IU/L (age 18–20 years) and ≥ 31 IU/L (age ≥ 21 years). Adjusted odds ratios (ORs) for ALT elevation were determined across exposure quartiles for 17 pollutant subclasses comprising 111 individual pollutants present with at least a 60% detection rate. Adjustments were made for age, race/ethnicity, sex, body mass index, poverty income ratio, and insulin resistance. Individual pollutants from subclasses associated with ALT elevation were subsequently analyzed.

**Results:**

The overall prevalence of ALT elevation was 10.6%. Heavy metals and polychlorinated biphenyls (PCBs) were associated with dose-dependent increased adjusted ORs for ALT elevation. Within these subclasses, increasing whole-blood levels of lead and mercury and increasing lipid-adjusted serum levels of 20 PCBs were individually associated with ALT elevation.

**Conclusions:**

PCB, lead, and mercury exposures were associated with unexplained ALT elevation, a proxy marker of NAFLD, in NHANES 2003–2004 adult participants.

The burden of liver disease has increased in the United States in parallel with the obesity epidemic, and some cases are believed to be due to nonalcoholic fatty liver disease (NAFLD) or its more advanced form, nonalcoholic steatohepatitis (NASH) (Cave et al. 2007). Serum alanine aminotransferase (ALT) is the most specific of the routinely used biomarkers for hepatocellular liver injury and disease in clinical medicine (Green and Flamm 2002). Currently, there is no serologic biomarker to confirm the diagnosis of NAFLD, but ALT elevation (above normal laboratory reference ranges) is the most common laboratory manifestation of NAFLD, and ALT elevation unexplained by viral hepatitis, ethanol, or iron overload has been used as a surrogate biomarker for NAFLD in the National Health and Nutrition Examination Survey (NHANES) (Clark 2006). Using this approach, Clark et al. (2003) reported that “unexplained ALT elevation” or “suspected NAFLD” was present in 5.4% of adult NHANES III (1988–1994) participants. Since the publication of that study, unexplained ALT elevation has been rapidly adopted by other authors to define subpopulations within NHANES with suspected NAFLD (Clark et al. 2003; Dunn et al. 2008; Liangpunsakul and Chalasani 2004, 2005).

High-level occupational exposures to industrial chemicals have been associated with liver diseases, including NAFLD (Cave et al. 2007; Cotrim et al. 1999, 2004). Recently, insulin resistance and toxicant-associated steatohepatitis (TASH), a form of NASH, were reported in nonobese chemical workers who had been highly exposed to vinyl chloride (Cave et al. 2010). However, the potential influence of low-level environmental pollution on liver disease and suspected NAFLD in the general population has not been determined. Recently, pollutant levels were measured in NHANES participants, and specific pollutants were found to be associated with insulin resistance and metabolic syndrome (Lee et al. 2007a, 2007b), although liver disease was not evaluated.

In the present study, we tested the hypothesis that environmental pollutants are dose-dependently associated with increased risk for ALT elevation and suspected NAFLD in the NHANES adult population.

## Materials and Methods

### Study design and participants

Adult participants from NHANES 2003–2004, the most recent NHANES for which pollutant data were posted at the time of analysis, were evaluated in this cross-sectional cohort study. NHANES is conducted by the National Center for Health Statistics (NCHS) to evaluate the health and nutrition of Americans, and it is designed to be nationally representative of the noninstitutionalized U.S. civilian population on the basis of a complex multistage probability sample (NCHS 2005). Approval for our analysis of the NHANES data was granted by the University of Louisville Institutional Review Board.

**Figure 1:**
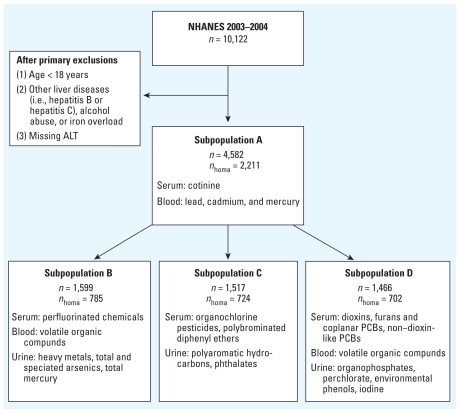
Schematic diagram depicting adult NHANES 2003–2004 subjects and pollutant subclasses analyzed. *n*_homa_ refers to the subset of subjects with HOMA-IR scores, which were required for data adjustment, and represents the maximum number of subjects available for each subpopulation for analysis by the primary model that adjusted for insulin resistance. The actual numbers used varied by analyte primarily because of missing response variables required for the other adjustments. The final numbers used for each analyte or chemical subclass are given in Tables 2–5 and in the Supplemental Material, Tables 2–5 (doi:10.1289/ehp.1002720). Volatile organic compounds were measured in selected subjects from subpopulations B and D.

For our study, we used the following exclusion criteria: age < 18 years, positive serum hepatitis B surface antigen, positive serum hepatitis C antibody, elevated transferrin saturation (> 60% for men and > 50% for women), and alcohol consumption ≥ 20 g/day for men and ≥ 10 g/day for women. Consistent with prior studies, these exclusions were used to identify suspected cases of NAFLD based on ALT elevation in the absence of other identifiable causes of liver disease (Clark et al. 2003). A total of 10,122 subjects were evaluated for eligibility, but after applying these exclusion criteria and eliminating another 643 subjects with missing ALT values, the final maximum sample size was 4,582 (Figure 1).

### Pollutants

All pollutant data posted by the NCHS before December 2008 were accessed and downloaded, which yielded 196 pollutants from 17 subclasses as categorized by the NCHS: serum perfluorinated compounds; urinary heavy metals; urinary total arsenic and speciated arsenics; urinary total (elemental plus inorganic) mercury; serum organochlorine pesticides; serum polybrominated diphenyl ethers (PBDEs); urinary polyaromatic hydrocarbons; urinary phthalates; serum polychlorinated dibenzo-*p*-dioxins (PCDDs), polychlorinated dibenzofurans (PCDFs), and coplanar polychlorinated biphenyls (PCBs); serum non–dioxin-like PCBs; urinary organophosphate insecticides; urinary perchlorate; urinary environmental phenols; urinary iodine; blood lead, mercury (total and inorganic), and cadmium; serum cotinine; and blood volatile organic compounds [for the full list of chemicals in each subclass, see Supplemental Material, Table 1 (doi:10.1289/ehp.1002720)]. An additional subclass, coplanar PCBs, was constructed by selecting only these chemicals from the broader “PCDDs, PCDFs, and coplanar PCBs” subclass. A second subclass for total PCBs was then created by combining the non–dioxin-like PCBs and coplanar PCBs subclasses.

All ALT and pollutant levels were measured in biologic samples collected on the same day from each individual participant. We evaluated only pollutants with a ≥ 60% detection rate [111 of 196 pollutants; see Supplemental Material, Table 1 (doi:10.1289/ehp.1002720)] to avoid bias in estimation for those pollutants with levels < the lower limit of detection (Lee et al. 2007a, 2007b). Concentrations of organic pollutants measured in serum (non–dioxin-like PCBs; dioxins, furans, coplanar PCBs; PBDEs; organochlorine pesticides) were lipid adjusted, and concentrations of pollutants measured in urine were adjusted for creatinine [Supplemental Material, Table 1 (doi:10.1289/ehp.1002720)] (Schwartz et al. 2003).

### Outcome variables and statistical methods

Serum ALT activity was measured by Collaborative Laboratory Services, LLC (Ottumwa, IA) for NHANES using the Beckman Synchron LX20 (Beckman Coulter, Brea, CA). Elevated ALT was defined as any value greater than this laboratory’s reference range, which was established from data obtained from healthy wellness participants as described in the NHANES Laboratory Procedure Manual (NCHS 2003–2004). Specifically, we used nonparametric techniques to estimate lower and upper bounds of reference ranges (2.5th and 97.5th percentiles) according to age and sex (Reed et al. 1971). ALT above the reference range was classified as elevated (men 18–20 years of age, ALT ≥ 37 IU/L; men ≥ 21 years old, ALT ≥ 48 IU/L; women 18–20 years of age, ALT ≥ 30 IU/L; women ≥ 21 years of age, ALT ≥ 31 IU/L). We determined the prevalence of ALT elevation in 4,582 subjects, and we used the chi-square test to determine statistically significant differences (*p* < 0.05) in ALT elevation and pollutant exposures according to sex, age, race/ethnicity, and body mass index (BMI).

Pollutant concentrations were classified according to a common scale that could be aggregated to assess cumulative exposures to multiple pollutants within a subclass. Specifically, we ranked each participant according to their measured concentration of each pollutant and summed the ranks of each one within a given subclass to determine their combined exposure (Lee et al. 2007a, 2007b). Ties were handled by assigning the minimum of the corresponding ranks to each participant, and participants with levels < the lower limit of detection (LLOD) for a pollutant were assigned the LLOD and ranked accordingly. For each pollutant subclass, subjects were stratified into quartiles by their cumulative exposure rank, with the first quartile representing subjects with the lowest levels. We estimated multivariate-adjusted odds ratios (ORs) for unexplained ALT elevation using logistic regression models with the first quartile as the reference group. Models were adjusted for age, race/ethnicity, and poverty income ratio (PIR). We also adjusted for both BMI and homeostasis model assessment of insulin resistance (HOMA-IR), because multiple pollutants have previously been associated with obesity and insulin resistance in NHANES (Lee et al. 2007a, 2007b). However, fasting glucose and insulin were measured in only a subset of NHANES participants, so only 2,211 subjects could be evaluated in this fashion. Further, although lead, cadmium, and mercury measurements were available for all 2,211 of these observations, other pollutant subclasses were measured only in subsets of this sample (perfluorinated chemicals, 785 subjects; organochlorine pesticides and polybrominated diethyl ethers, 724 subjects; dioxins, furans, coplanar PCBs, and non–dioxin-like PCBs, 702 subjects; see Figure 1).

Associations with individual chemicals were estimated if trend tests for the association between the entire subclass and elevated ALT were statistically significant. Subjects with detectable levels of individual pollutants were ranked, placed into quartiles, and compared with a reference group consisting of individuals with levels < LLOD or individuals in the first quartile of exposure (if none of the subjects had levels < LLOD, or if none of the subjects with levels < LLOD had elevated ALT). In the latter situation, subjects with levels < LLOD were still used in calculating the trend statistic for association of exposure level with elevated ALT.

We performed all statistical analyses using SURVEYFREQ and SURVEYLOGISTIC in SAS (version 9.1; SAS Institute Inc., Cary, NC). Estimates were adjusted for age, race/ethnicity, and PIR rather than using sample weights, which is regarded as a good compromise between efficiency and bias (Graubard and Korn 1999; Korn and Graubard 1991). Trend tests for the association between pollutants (both individual and cumulative for a subclass) were performed by modeling ordinal variables with integer scores assigned to each quartile and to the group of individuals with levels < LLOD, when appropriate [see Supplemental Material, Statistical Methods (doi:10.1289/ehp.1002720)]. *p*-Values were determined, both with (adjusted *p*_trend_) and without (*p*_trend_), for multiple comparisons using the false discovery rate method of Benjamini and Hochberg (1995), and both *p*-values are reported because adjustment for multiple comparisons has not consistently been performed in the other studies (Lee et al. 2007a, 2007b). Adjustments for multiple comparisons were done separately for subclass analyses and for analyses of individual pollutants within subclasses. We used a *p*-value ≤ 0.05 to indicate statistical significance.

## Results

### Demographic information

The full study sample included slightly more women than men (Table 1). The mean age (and corresponding SD) was 47.2 ± 21.2 years (range, 18–85 years). Non-Hispanic whites accounted for 72.3% of the population. Body weights, as defined by National Institutes of Health (1998) guidelines, were fairly evenly distributed between normal weight, overweight, and obese, with very few subjects being underweight (1.7%).

### Prevalence of unexplained ALT elevation and PCB exposure

Of the 4,582 adult subjects remaining after applying the exclusion criteria, 436 had unexplained ALT elevation (i.e., suspected NAFLD), which corresponds to 10.6% of the U.S. adult population or 19.4 million people (after accounting for NHANES sampling weights). ALT elevation was more common in women than in men (11.9% vs. 9.2%; *p* = 0.020) (Table 1). ALT elevation was more common in Hispanics than in non-Hispanic whites (18.6% vs. 10.0%), whereas non-Hispanic blacks had a lower prevalence of ALT elevation (5.6%; *p* = 0.001). ALT elevation was most prevalent during the fifth and sixth decades and was more prevalent in overweight and obese participants than in normal-weight participants (10.7%, 15.7%, and 5.1%, respectively; *p* = 0.001).

Older age and non-Hispanic black race, but not BMI or sex, were significantly associated with total PCB levels in the highest quartile (Table 1). Age had the most pronounced association: 71.7% of participants age ≥ 70 years had PCB levels in the highest quartile, compared with only 2.2% of subjects < 30 years of age (*p* < 0.001). Non-Hispanic blacks (29.2%) were more likely to be in the highest quartile of total cumulative PCB exposure than were non-Hispanic whites (21.7%) and Hispanics (8.2%; *p* = 0.002).

### Pollutant subclass results

We estimated significant positive trends for adjusted ORs for 3 of the 17 NHANES pollutant subclasses investigated (Table 2). Specifically, the adjusted ORs and 95% confidence intervals (CIs) for the highest versus lowest quartiles of exposure were for serum dioxins, furans, and coplanar PCBs, 5.8 (95% CI, 1.1–30.2; *p*_trend_ = 0.024); for serum non–dioxin-like PCBs, 4.5 (95% CI, 2.0–10.0; *p*_trend_ < 0.001); and for blood lead, mercury, and cadmium, 1.6 (95% CI, 1.1–2.3; *p*_trend_ = 0.015). After adjusting for multiple comparisons, the trend test for non–dioxin-like PCBs remained statistically significant (*p*_trend-adj_ = 0.001). In general, results were comparable when estimated without adjustment for BMI or HOMA-IR [see Supplemental Material, Tables 3 and 4, respectively (doi:10.1289/ehp.1002720)], although trend tests for associations with creatinine-adjusted urine polyaromatic hydrocarbons and serum lipid-adjusted PBDEs indicated significant positive and negative trends in associations with ALT based on models without adjustment for HOMA-IR.

Significant positive trends were also evident for associations with coplanar PCBs and total PCBs (Table 2). The highest quartile of cumulative coplanar PCB exposure, compared with the lowest quartile, was associated with a significantly increased adjusted OR for ALT elevation of 7.6 (95% CI, 2.8–20.7; *p*_trend-adj_ < 0.001). Likewise, the adjusted OR for the highest versus lowest quartile of exposure to cumulative total PCB subclass (coplanar PCBs plus non–dioxin like PCBs) was 4.3 (95% CI, 1.8–10.1; *p*_trend-adj_ = 0.01).

### Individual pollutant results

We also estimated associations with 45 individual pollutants from subclasses with significant trend tests. Blood lead (99.6%) and total mercury (92.5%) had extremely high detection rates and were positively associated with ALT elevation, but cadmium exposure was not associated with ALT elevation (Table 3).

Ten coplanar PCBs were present at detection rates ranging from 68% to 100%, and nine of these were positively associated with elevated ALT (Table 4). However, none of the four PCDDs or three PCDFs was associated with ALT elevation [see Supplemental Material, Table 5 (doi:10.1289/ehp.1002720)]. Twenty-five non–dioxin-like PCBs were present, with detection rates ranging from 65.5% to 100%, and 11 of these were positively associated with ALT elevation with significant trends (Table 5).

## Discussion

The prevalence of ALT elevation unexplained by viral hepatitis, hemochromatosis, or alcoholism (i.e., suspected NAFLD) was 10.6% in NHANES 2003–2004, which was nearly double the prevalence (5.4%) reported by a study of NHANES 1988–1994 adult participants that used similar exclusion criteria and a similar ALT reference range (Clark et al. 2003). As in our study, Clark et al. (2003) also noted that ALT elevation was associated with BMI, Hispanic ethnicity, and middle age. The observed increase in the prevalence of ALT elevation from NHANES 1988–1994 to NHANES 2003–2004 is consistent with the growing burden of obesity and NAFLD.

Because liver biopsy was not performed in NHANES, we used unexplained ALT elevation as a proxy measure of liver disease and NAFLD and identified several ubiquitous environmental pollutants that were dose-dependently associated with suspected NAFLD, including lead, mercury, and PCBs. Although levels of many pollutants are decreasing in the environment, PCB, lead, and mercury exposures remain problematic. For example, even though PCBs were banned in 1977, 100% of subjects in this study had detectable PCB levels.

Diet-induced obesity probably plays the primary role in the pathogenesis of most cases of NAFLD (Cave et al. 2007), but nutrient–toxicant interactions and genetic susceptibility to environmental pollution may be important cofactors, which we did not address in this study. Data from our group and others suggest that diet-induced obesity and fatty liver decrease antioxidant defenses and impair xenobiotic metabolism and disposition, which could sensitize the liver to chemical injury (Fisher et al. 2009a, 2009b; Kirpich et al. in press). Further complicating this issue, lead, mercury, and coplanar PCBs concentrate within the liver, whereas non–dioxin-like PCBs concentrate in adipose tissue and possibly in steatotic (fatty) livers [Klein et al. 1972; Mudipalli 2007; National Toxicology Program (NTP) 2006a]. Therefore, tissue levels may not always correlate with serum levels. However, it is important to recognize that multiple animal studies demonstrate that PCBs and methylmercury (MeHg) exposures induce fatty liver, even in the absence of diet-induced obesity (Chang and Yamaguchi 1974; Desnoyers and Chang 1975b; Lin et al. 1996; NTP 2006a, 2006b, 2006c). Although lead has been associated with hepatic hyperplasia and not NAFLD, to our knowledge lead and diet-induced obesity coexposures have not been performed in animal models (Mudipalli 2007). The results of these aforementioned studies lend biologic plausibility to the hypothesis that lead, mercury, and PCBs may play a previously unsuspected role in the pathogenesis of some cases of suspected NAFLD.

PCBs are polyhalogenated aromatic hydrocarbons that consist of up to 10 chlorine atoms attached to a biphenyl group. About 130 of the 209 theoretical PCB congeners were manufactured between 1929 and 1977 as mixtures and were sold as a function of chlorine content. For example, Monsanto marketed Aroclors 1221, 1231, and 1242 up to 1268, which contain, respectively, 21%, 31%, and 42% to 68% chlorine by weight. Aroclors were used in multiple industrial applications and were components in dielectric insulating fluids for transformers and capacitors, hydraulic fluids, plastics, and paints. An estimated 1.3 million tons of PCBs were manufactured almost exclusively (97%) in the northern hemisphere (Breivik et al. 2002). Although PCBs have been banned in the United States for > 30 years, their high thermodynamic stability makes them resistant to biodegradation and thus persistent organic pollutants. More highly chlorinated PCBs tend to be metabolized and eliminated more slowly, and the PCBs identified in biologic samples in this study were indeed the more highly chlorinated varieties.

From a mechanistic standpoint, a PCB’s structure determines its ability to interact with nuclear receptors. Like PCDDs and PCDFs, coplanar PCBs are aryl hydrocarbon receptor (AhR) agonists, and PCB-126 accounts for 52% of the toxic equivalency of dioxin-like PCBs in human tissues (NTP 2006b; Safe 1993). In comparison, some non–dioxin-like PCBs such as PCB-153 do not activate AhR but may be constitutive androstane receptor agonists (Dean et al. 2002). Animal studies demonstrate that non–dioxin-like PCBs such as PCB-153 are concentrated most heavily within the adipose tissue because of their high lipid solubility (NTP 2006b). Coplanar PCBs, such as PCB 126, despite high lipid solubility, paradoxically concentrate primarily within the liver (NTP 2006b). In our study, both types of PCBs, including PCB-126 and PCB-153, were dose-dependently associated with ALT elevation.

Extensive animal studies conducted by the NTP and others have defined a role for PCBs in liver disease. The NTP has performed 2-year toxicity studies on PCB-126 and PCB-153 in female Harlan Sprague-Dawley rats (NTP 2006a, 2006b, 2006c). These studies demonstrated that the liver was the principal target organ for these compounds. Both benign (toxic hepatopathy, including steatosis) and malignant (hepatocellular carcinoma and cholangiocarcinoma) liver lesions were observed at high frequencies in a dose-dependent fashion, particularly in animals treated with PCB-126 alone or combined with PCB-153. Importantly, both of these PCBs were associated with human ALT elevation in our study. Hennig et al. (2005) demonstrated that PCB-77 exacerbated high-fat-diet (corn oil)–induced hepatic steatosis in mice and increased hepatic gene expression of genes involved in apoptosis, inflammation, and oxidative stress. However, this particular coplanar PCB was not measured in NHANES 2003–2004. In contrast to animal studies, human data on PCBs in liver disease are lacking. However, in Taiwan, 13 years after the “Yucheng” incident where cooking oil was contaminated by PCBs, the mortality rate due to cirrhosis was 2.7 times higher than expected (Yu et al. 1997).

Whole-blood total mercury, present in 92.5% of subjects, but not urinary total (inorganic plus elemental) mercury, was dose-dependently associated with ALT elevation and suspected NAFLD. These results suggest that the organic form of mercury was associated with liver disease. MeHg is the principal form of organic mercury historically associated with organ toxicity. Since the 1950s outbreak of Minamata disease (MeHg intoxication) in a Japanese fishing village, MeHg has been recognized as one of the most hazardous environmental pollutants. Coal-fired power plants have been identified as the primary source of current mercury emissions, and atmospheric mercury may be converted into MeHg in water-body sediment and subsequently enter the aquatic food chain and bioaccumulate in fish (Charnley 2006). The primary route of human MeHg exposure is consumption of contaminated fish and shellfish, and PCB coexposure may occur (Charnley 2006). MeHg has well-characterized toxic effects on the human nervous system, developing fetus, and kidney (Charnley 2006).

Despite the fact that MeHg concentrates considerably within the liver because of enterohepatic recirculation, few animal studies have examined the potential role of MeHg in liver disease. However, acute and chronic toxicity studies conducted in rats and cats demonstrated that mercury exposure resulted in the depletion of body fat, the development of centrilobular hepatic steatosis, an increase in lipid peroxidation products, the proliferation of the endoplasmic reticulum, and floccular degeneration of the mitochondria with extrusion of diseased organelles into the sinusoidal space (Chang and Yamaguchi 1974; Desnoyers and Chang 1975a, 1975b; Klein et al. 1972; Lin et al. 1996). Many of these changes were irreversible after exposure to MeHg was discontinued. The primary mechanism of MeHg hepatotoxicity may be related to its high affinity for sulfhydryl residues and consequent poisoning of cysteine-containing proteins and glutathione depletion (Lin et al. 1996). Previous human epidemiological studies have inconsistently linked mercury contamination in Japanese fishing villages to increased liver-related mortality in villagers (Futatsuka et al. 1987, 1992, 2005).

With a detection rate of 99.6%, lead exposure was nearly universal in adult NHANES subjects. In contrast to PCBs and MeHg, lead hepatotoxicity is relatively well recognized and was recently reviewed (Mudipalli 2007). Lead exposure most commonly occurs through the respiratory or gastrointestinal system. Regardless of the route of exposure, the liver is the largest lead repository in the body (Mudipalli 2007). The pathologic liver lesion of lead exposure has been termed “lead-induced hepatic hyperplasia,” but hepatic steatosis has not been reported. Multiple molecular events have been described in association with lead-induced hepatic hyperplasia. Oxidative stress, proinflammatory cytokine production and sensitivity, and liver and serum cholesterol levels were all increased by lead (Aykin-Burns et al. 2003; Honchel et al. 1991; Kojima et al. 2004; Milosevic and Maier 2000; Sandhir and Gill 1995).

Several potential problems are inherent to the design of this study. The exact specificity of ALT for liver disease in NHANES is unknown because liver biopsies were not performed. However, ALT should be relatively specific, because the incidence of myopathy, the most important extrahepatic source of ALT, is likely low in the general population (Green and Flamm 2002). In contrast, at the reference range used in this study, the sensitivity of ALT is likely lower than its specificity. In fact, some authors have suggested using lower laboratory cutoffs to gain more sensitivity (Prati et al. 2002; Ruhl and Everhart 2009). Importantly, ALT may be normal in NAFLD, and this appears to be an even bigger problem in fatty liver and TASH due to some industrial chemicals (Brautbar and Williams 2002; Cave et al. 2010). Therefore, low-level environmental pollution may pose an even greater risk for liver disease in the general U.S. population than suggested by the results of this study. Lastly, the cross-sectional study design of NHANES cannot determine the direction of causation for the identified associations between environmental pollutant levels and elevated ALT. It is possible that these pollutant concentrations may be elevated because of the presence of liver disease or another predisposing factor for elevated ALT, rather than the risk of elevated ALT being increased because of elevated pollutant levels.

The pollutant subclassifications created by NHANES, although generally reasonable, may not always have the most biologic relevance. For example, heavy metals were grouped differently according to the method of measurement (blood or urine). Given the large number of measured pollutants, looking at all possible groupings of pollutants and mixtures of subgroups was not practical. However, we created new PCB subclasses for coplanar and total PCBs because these molecules were consistently associated with ALT elevation.

Regarding PCBs, NHANES reported levels for only a quarter of the 130 manufactured PCB congeners, so it must be acknowledged that this study did not actually model the effects of total lipid-adjusted serum PCB burden. However, because PCBs were sold in mixtures, it is likely that subjects high in the measured PCBs would also be high in the others. As with all other subclasses, members of the tetrachlorodibenzo-*p*-dioxin, PCDDs and coplanar PCB subclasses were ranked by serum concentration, which did not account for their toxic equivalency factors. This method allowed us to combine the coplanar PCB and non–dioxin-like PCB subclasses to form the total PCB subclass. However, AhR-dependent hepatotoxicities could be examined by alternate models. Also, although ranking individuals on the basis of exposure levels rather than modeling serum pollutant levels directly allowed us to compare results between individual pollutants and pollutant subclasses, this approach limits comparisons with other study populations.

## Conclusion

PCBs, lead, and mercury are present in nearly all U.S. adults. These common pollutants are associated with significant dose-dependent increased ORs for ALT elevation in subjects whose ALT elevations were not explained by viral hepatitis, hemochromatosis, or alcohol abuse. These results suggest a possible association between low-level environmental pollution and the development of liver disease and suspected NAFLD. Future studies should be performed to confirm the potential role of these environmental pollutants in NAFLD.

## Figures and Tables

**Table 1:** Prevalence of unexplained ALT elevation (suspected NAFLD) and association with elevated total PCB levels in adult NHANES 2003–2004, by demographic groups.

Demographic variable	Population distribution (%)	Prevalence of unexplained ALT elevation			PCBs > 75th percentile^a^		
		Percent	SE	*p*-Value	Percent	SE	*p*-Value
Sex				0.020			0.182

Male	47.8	9.2	0.8		18.5	2.1	
Female	52.2	11.9	0.5		22.8	1.8	

Race/ethnicity				< 0.001			0.002

Non-Hispanic White	72.3	10.0	0.5		21.7	1.4	
Non-Hispanic Black	10.8	5.6	0.8		29.2	2.9	
Hispanic	11.7	18.6	1.5		8.2	2.2	
Other	5.1	10.9	2.9		19.3	8.3	

Age (years)				0.005			< 0.001

< 30	21.5	9.1	1.2		2.2	1.2	
30–40	19.5	11.8	1.3		4.2	1.5	
40–50	20.4	13.2	1.4		12.0	2.1	
50–60	16.0	13.0	1.2		24.6	5.1	
60–70	10.5	10.6	2.0		45.0	5.2	
≥ 70	12.1	3.7	0.9		71.7	4.4	

BMI (kg/m^2^)				< 0.001			0.525

< 18.5	1.7	7.1	3.1		17.7	9.9	
18.5–24.9	31.5	5.1	0.8		17.6	1.9	
25–29.9	34.3	10.7	1.3		23.1	2.7	
≥ 30	32.5	15.7	1.0		21.6	2.4	

SE, standard error of the mean. *p*-Values were calculated by chi-square tests.

a Percentage of subjects within a demographic group having lipid-adjusted serum total PCB levels in the highest exposure quartile.

**Table 2:** Adjusted ORs^a^ (95% CIs) for ALT elevation by exposure quartile for pollutant subclasses in adults, NHANES 2003–2004.

Pollutant subclass	n	Pollutants screened/analyzed^b^	Quartile [cases/total, OR (95% CI)]				*p*-Value	
			First	Second	Third	Fourth	Trend	Adjusted trend^c^
Coplanar PCBs (serum)	535	10/9	12/133	12/137	18/141	15/129	< 0.001	< 0.001
			Referent	2.2 (0.9–5.2)	4.4 (1.8–10.5)	7.6 (2.8–20.7)		

Non–dioxin-like PCBs (serum)	532	26/25	18/125	9/145	15/131	17/131	< 0.001	0.001
			Referent	0.8 (0.3–2.0)	2.4 (1.1–5.2)	4.5 (2.0–10.0)		

Total PCBs (serum)	535	36/34	18/119	8/129	15/138	15/119	0.002	0.010
			Referent	0.8 (0.3–2.3)	2.2 (0.9–5.3)	4.3 (1.8–10.1)		

Dioxins, furans, coplanar PCBs (serum)	535	29/17	13/138	12/136	17/135	13/126	0.024	0.137
			Referent	1.7 (0.7–4.4)	3.6 (1.2–10.8)	5.8 (1.1–30.2)		

Lead, cadmium, mercury (blood)	2,051	4/3	57/520	45/505	49/518	54/508	0.015	0.126
			Referent	0.9 (0.6–1.4)	1.1 (0.8–1.7)	1.6 (1.1–2.3)		

Environmental phenols (urine)	643	4/3	19/145	22/162	11/165	17/171	0.333	0.811
			Referent	1.2 (0.5–2.7)	0.5 (0.2–1.3)	0.9 (0.4–2.1)		

Polyaromatic hydrocarbons (urine)	563	21/10	14/147	10/139	15/142	17/135	0.335	0.811
			Referent	0.8 (0.3–2.6)	1.0 (0.4–2.7)	1.6 (0.6–4.6)		

PBDEs (serum)	614	11/6	20/169	19/149	18/149	13/147	0.354	0.811
			Referent	1.0 (0.5–1.9)	0.9 (0.4–1.9)	0.7 (0.3–1.5)		

Volatile organic compounds (blood)	433	33/6	10/108	16/97	13/111	14/117	0.427	0.811
			Referent	2.0 (0.8–4.7)	1.3 (0.6–2.9)	1.6 (0.8–3.4)		

Perfluorinated compounds (serum)	694	12/4	18/189	13/169	12/167	15/169	0.460	0.811
			Referent	0.8 (0.3–2.1)	1.0 (0.3–3.0)	1.6 (0.5–5.1)		

Perchlorate (urine)	638	1/1	13/161	17/163	21/166	18/148	0.533	0.811
			Referent	1.2 (0.6–2.3)	1.4 (0.7–2.8)	1.2 (0.5–2.9)		

Phthalates (urine)	655	13/9	23/177	17/158	13/150	19/170	0.553	0.811
			Referent	0.9 (0.3–2.3)	0.6 (0.3–1.6)	0.9 (0.5–1.6)		

Organophosphate insecticides (urine)	631	6/4	26/159	10/150	13/157	18/144	0.561	0.811
			Referent	0.4 (0.2–0.7)	0.5 (0.2–1.0)	0.8 (0.5–1.3)		

Iodine (urine)	645	1/1	18/158	16/160	18/180	16/147	0.572	0.811
			Referent	0.7 (0.4–1.2)	0.7 (0.4–1.2)	0.8 (0.4–1.5)		

Total and speciated arsenics (urine)	710	8/3	15/188	14/187	17/170	13/165	0.725	0.901
			Referent	0.8 (0.3–2.0)	1.3 (0.7–2.3)	0.9 (0.5–1.9)		

Cotinine (serum)	2,050	1/1	54/527	49/515	58/504	43/504	0.783	0.901
			Referent	0.9 (0.6–1.4)	1.3 (0.9–1.9)	1.0 (0.6–1.7)		

Heavy metals (urine)	709	12/9	15/184	17/197	11/179	16/149	0.807	0.901
			Referent	0.9 (0.4–1.9)	0.5 (0.2–1.2)	1.1 (0.5–2.3)		

Organochlorine pesticides (serum)	587	13/8	15/136	15/140	21/155	12/156	0.848	0.901
			Referent	0.9 (0.5–1.6)	1.5 (0.5–4.2)	0.9 (0.2–3.7)		

Total mercury (urine)	707	1/1	11/180	20/167	11/169	16/191	0.918	0.918
			Referent	2.3 (0.8–6.8)	0.9 (0.3–2.8)	1.5 (0.5–4.7)		

Detectable values of each pollutant were individually ranked, and the rank orders of the individual pollutants in each subclass were summed to arrive at the subclass value. All nondetectable values were ranked as one. The summary values were categorized by cutoff points of 25th, 50th, and 75th values of the sum of ranks.

a ORs were adjusted for age, sex, race, PIR, HOMA-IR, and BMI.

b In any given pollutant subclass, only chemicals with at least a 60% detection rate were included in the analysis.

c Additionally adjusted for multiple comparisons.

**Table 3:** Adjusted ORs^a^ (95% CIs) for ALT elevation by exposure quartile for lead, cadmium, and mercury in adults, NHANES 2003–2004.

Pollutant	Detection rate (%)	Not detectable (cases/total)	Detectable [median concentration, cases/total, OR (95% CI)]				*p*-Value	
			First quartile	Second quartile	Third quartile	Fourth quartile	Trend	Adjusted trend^b^
Lead (μg/dL)	99.6		0.80	1.30	1.90	3.30	0.006	0.014
		0/6	55/579	48/494	53/498	49/474		
			Referent	1.2 (0.9–1.7)	1.5 (1.0–2.1)	1.6 (1.1–2.3)		
Mercury, total (μg/L)	92.5		0.40	0.80	1.40	3.10	0.010	0.014
		12/158	40/500	64/540	50/395	39/458		
		Referent	1.1 (0.7–1.8)	2.0 (1.3–3.2)	2.2 (1.4–3.5)	1.6 (1.1–2.4)		
Cadmium (μg/L)	82.8		0.30	0.40	0.60	1.10	0.503	0.503
		38/345	73/672	21/257	38/406	35/371		
		Referent	1.1 (0.7–1.7)	0.9 (0.6–1.4)	1.2 (0.8–1.7)	1.2 (0.7–2.0)		

a ORs were adjusted for age, sex, race, PIR, HOMA-IR, and BMI.

b Additionally adjusted for multiple comparisons.

**Table 4:** Adjusted ORs^a^ (95% CIs) for ALT elevation by exposure quartile for coplanar PCBs in adults, NHANES 2003–2004.

Pollutant (lipid adjusted)	Detection rate (%)	Not detectable (cases/total)	Detectable [median concentration, cases/total, OR (95% CI)]				*p*-Value	
			First quartile	Second quartile	Third quartile	Fourth quartile	Trend	Adjusted trend^b^
PCB-28 (ng/g)	100.0		2.75	4.28	5.75	8.77	0.740	0.839
			17/140	12/151	14/141	18/139		
			Referent	0.5 (0.2–1.4)	0.8 (0.4–1.6)	1.0 (0.4–2.3)		

PCB-66 (ng/g)	98.9		0.74	1.18	1.67	3.00	0.003	0.011
		0/8	12/135	8/150	25/154	18/134		
			Referent	0.6 (0.2–1.5)	2.9 (1.6–5.4)	1.9 (0.9–4.0)		

PCB-74 (ng/g)	100.0		1.76	3.32	6.82	17.88	< 0.001	0.004
			14/142	15/150	19/157	15/132		
			Referent	2.2 (1.0–5.1)	3.0 (1.5–6.0)	6.0 (2.4–14.9)		

PCB-105 (ng/g)	98.0		0.52	0.93	1.51	4.46	0.015	0.031
		0/11	13/142	12/150	21/144	17/131		
			Referent	1.2 (0.5–3.0)	2.8 (1.2–6.5)	3.4 (1.1–10.9)		

PCB-118 (ng/g)	100.0		2.30	4.26	8.16	22.80	0.006	0.016
			12/142	14/158	22/144	15/135		
			Referent	1.8 (0.7–4.8)	3.8 (1.3–11.1)	4.4 (1.4–13.7)		

PCB-126 (pg/g)	94.8		8.70	13.80	22.00	50.50	< 0.001	< 0.001
		0/33	9/134	12/127	22/146	18/123		
			Referent	1.5 (0.6–3.5)	3.3 (1.5–7.2)	4.3 (2.0–9.4)		

PCB-156 (ng/g)	91.7		0.90	2.74	6.11	12.40	< 0.001	0.004
		4/49	15/130	14/131	16/139	14/130		
		Referent	1.8 (0.6–5.5)	3.4 (1.3–8.7)	5.0 (1.5–17.2)	9.4 (2.5–36.2)		

PCB-157 (ng/g)	74.9		0.41	1.00	1.79	3.30	0.006	0.016
		15/152	11/111	17/105	8/100	11/105		
		Referent	1.5 (0.8–2.8)	4.1 (1.8–9.2)	2.1 (0.6–7.6)	7.1 (2.2–22.4)		

PCB-167 (ng/g)	68.0		0.50	1.10	1.93	3.81	0.003	0.011
		19/188	9/103	12/104	11/90	11/90		
		Referent	0.9 (0.4–2.2)	2.2 (1.0–4.8)	2.7 (1.0–7.0)	5.0 (1.9–13.3)		

PCB-169 (pg/g)	70.3		7.50	13.50	22.40	39.65	0.032	0.061
		14/164	15/107	11/94	17/111	4/89		
		Referent	2.4 (0.9–7.0)	3.5 (1.3–9.7)	5.0 (1.8–14.0)	2.4 (0.4–12.8)		

Coplanar PCBs were measured in serum and are reported as lipid-adjusted values.

a ORs were adjusted for age, sex, race, PIR, HOMA-IR, and BMI.

b Additionally adjusted for multiple comparisons.

**Table 5:** Adjusted ORs^a^ (95% CIs) for ALT elevation by exposure quartile for non–dioxin-like PCBs in adults, NHANES 2003–2004.

Pollutant (lipid adjusted)	Detection rate (%)	Not detectable (cases/total)	Detectable [median concentration, cases/total, OR (95% CI)]				*p*-Value	
			First quartile	Second quartile	Third quartile	Fourth quartile	Trend	Adjusted trend^b^
PCB-44 (ng/g)	99.9		1.00	1.70	2.49	4.00	0.587	0.734
			17/146	9/133	21/162	16/136		
			Referent	0.6 (0.2–2.1)	1.2 (0.7–2.0)	1.0 (0.4–2.7)		

PCB-49 (ng/g)	99.4		0.63	1.10	1.60	2.55	0.779	0.928
		1/3	17/146	10/131	17/155	18/139		
		Referent	0.2 (0.0–3.2)	0.1 (0.0–2.1)	0.2 (0.0–2.4)	0.2 (0.0–4.0)		

PCB-52 (ng/g)	100.0		1.27	2.17	3.40	5.40	0.571	0.734
			18/150	11/128	11/159	23/143		
			Referent	0.7 (0.3–1.8)	0.5 (0.2–1.0)	1.5 (0.6–3.8)		

PCB-87 (ng/g)	83.5		0.57	0.90	1.20	2.01	0.354	0.520
		11/95	16/120	5/118	13/119	18/128		
		Referent	0.9 (0.5–1.7)	0.3 (0.1–0.7)	0.9 (0.5–1.7)	1.3 (0.6–2.7)		

PCB-99 (ng/g)	100.0		1.73	3.15	5.40	12.90	0.149	0.286
			16/139	13/156	19/146	15/135		
			Referent	1.1 (0.5–2.6)	2.1 (0.6–7.9)	2.4 (0.7–8.9)		

PCB-101 (ng/g)	96.6		0.76	1.42	2.20	4.00	0.210	0.336
		2/18	14/148	12/127	12/144	23/144		
		Referent	0.3 (0.1–1.8)	0.3 (0.1–1.5)	0.4 (0.1–2.1)	0.7 (0.1–3.9)		

PCB-110 (ng/g)	98.4		0.51	1.00	1.59	3.03	0.427	0.593
		1/7	17/152	12/138	12/146	21/136		
		Referent	0.5 (0.1–5.4)	0.4 (0.0–4.4)	0.4 (0.0–4.4)	0.8 (0.1–6.9)		

PCB-138 and PCB-158 (ng/g)	100.0		4.58	11.07	25.10	57.98	0.001	0.009
			15/148	14/140	15/159	19/133		
			Referent	1.9 (0.8–4.1)	2.5 (1.0–6.0)	6.7 (2.1–21.5)		

PCB-146 (ng/g)	99.2		0.61	1.70	4.00	8.90	0.004	0.019
		0/7	14/140	18/148	14/147	17/139		
			Referent	2.2 (1.0–4.5)	2.7 (1.1–6.9)	6.8 (1.8–25.5)		

PCB-149 (ng/g)	95.8		0.31	0.52	0.79	1.33	0.215	0.336
		4/19	13/135	12/133	11/160	21/129		
		Referent	0.2 (0.1–0.7)	0.3 (0.1–0.9)	0.2 (0.0–0.8)	0.6 (0.2–2.0)		

PCB-151 (ng/g)	80.2		0.19	0.30	0.41	0.79	0.030	0.068
		11/115	14/113	8/112	10/116	19/121		
		Referent	1.1 (0.3–3.6)	0.8 (0.3–2.1)	1.0 (0.4–2.3)	2.6 (1.2–5.8)		

PCB-153 (ng/g)	100.0		5.59	15.18	35.01	75.55	0.006	0.023
			17/145	13/147	14/153	19/136		
			Referent	1.5 (0.6–3.6)	2.3 (0.7–7.4)	7.2 (1.7–29.9)		

PCB-170 (ng/g)	99.2		1.40	4.56	11.00	22.70	0.015	0.042
		0/3	15/147	18/141	17/151	13/137		
			Referent	2.1 (1.0–4.3)	3.1 (1.1–8.7)	4.4 (1.3–14.4)		
PCB-172 (ng/g)	77.1		0.42	1.10	1.99	3.73	0.007	0.023
		13/131	15/120	12/107	13/119	10/100		
		Referent	1.4 (0.7–3.1)	2.1 (0.8–5.4)	2.7 (0.9–8.1)	3.4 (1.2–9.7)		

PCB-177 (ng/g)	89.3		0.53	1.30	2.70	6.00	< 0.001	< 0.001
		7/60	9/123	16/144	15/124	16/128		
		Referent	0.7 (0.4–1.3)	2.0 (1.0–3.9)	4.2 (1.7–10.4)	6.5 (2.8–15.3)		

PCB-178 (ng/g)	85.9		0.40	1.20	2.44	4.86	0.014	0.042
		7/70	16/130	13/133	17/127	10/118		
		Referent	1.7 (0.8–3.9)	2.1 (1.0–4.6)	4.6 (1.4–15.3)	4.8 (1.3–17.4)		

PCB-180 (ng/g)	99.8		3.40	11.90	29.40	66.51	0.206	0.336
			16/145	17/147	19/151	11/137		
			Referent	1.5 (0.7–3.2)	2.5 (0.9–7.3)	2.4 (0.6–10.4)		

PCB-183 (ng/g)	93.6		0.60	1.40	2.99	6.37	0.017	0.042
		2/33	16/131	17/150	11/133	17/132		
		Referent	2.4 (0.4–15.4)	4.0 (0.6–26.8)	3.1 (0.4–23.2)	7.8 (0.9–63.9)		

PCB-187 (ng/g)	99.2		1.10	3.40	8.33	18.40	< 0.001	0.002
		0/4	13/136	19/158	14/142	17/138		
			Referent	2.8 (1.6–5.0)	4.6 (1.6–13.3)	10.5 (3.2–34.6)		

PCB-194 (ng/g)	87.8		1.00	3.57	7.87	16.57	0.881	0.958
		9/68	17/125	13/123	15/137	9/114		
		Referent	1.2 (0.6–2.5)	1.0 (0.4–2.4)	1.1 (0.4–2.7)	1.2 (0.3–4.6)		

PCB-195 (ng/g)	65.5		0.60	1.40	2.33	4.23	0.862	0.958
		26/198	9/97	13/93	6/79	9/93		
		Referent	0.6 (0.2–1.6)	1.3 (0.5–3.4)	0.6 (0.2–1.8)	1.2 (0.5–2.9)		

PCB-196 and PCB-203 (ng/g)	93.6		0.80	2.60	5.92	12.50	< 0.001	0.002
		2/38	18/133	13/139	16/138	14/130		
		Referent	3.6 (0.9–13.8)	4.1 (1.1–16.0)	8.2 (1.7–39.3)	14.7 (3.3–65.3)		

PCB-199 (ng/g)	92.8		0.81	3.00	7.34	16.79	0.093	0.194
		4/40	19/127	12/149	17/129	11/128		
		Referent	1.9 (0.9–4.2)	1.2 (0.7–2.1)	2.5 (1.2–5.3)	3.3 (0.9–12.4)		

PCB-206 (ng/g)	96.9		0.50	1.60	4.40	10.90	0.971	0.982
		2/15	20/148	13/141	20/140	8/131		
		Referent	1.0 (0.4–2.6)	0.8 (0.3–2.3)	1.3 (0.6–3.2)	0.9 (0.3–2.2)		

PCB-209 (ng/g)	96.4		0.40	0.93	2.73	8.79	0.982	0.982
		0/18	23/147	15/142	14/134	10/131		
			Referent	0.9 (0.4–2.0)	0.8 (0.4–1.8)	1.1 (0.3–3.9)		

Non–dioxin-like PCBs were measured in serum and are reported as lipid-adjusted values.

a ORs were adjusted for age, sex, race/ethnicity, PIR, HOMA-IR, and BMI.

b Additionally adjusted for multiple comparisons.
